# Detecting Symptom Errors in Neural Machine Translation of Patient Health Information on Depressive Disorders: Developing Interpretable Bayesian Machine Learning Classifiers

**DOI:** 10.3389/fpsyt.2021.771562

**Published:** 2021-10-21

**Authors:** Wenxiu Xie, Meng Ji, Mengdan Zhao, Tianqi Zhou, Fan Yang, Xiaobo Qian, Chi-Yin Chow, Kam-Yiu Lam, Tianyong Hao

**Affiliations:** ^1^Department of Computer Science, City University of Hong Kong, Kowloon, Hong Kong, SAR China; ^2^School of Languages and Cultures, The University of Sydney, Darlington, NSW, Australia; ^3^Independent Researcher, Sichuan, China; ^4^School of Computer Science, South China Normal University, Guangzhou, China

**Keywords:** depressive disorder, mental health, neural machine translation (NMT), translation error detection, machine learning application

## Abstract

**Background:** Due to its convenience, wide availability, low usage cost, neural machine translation (NMT) has increasing applications in diverse clinical settings and web-based self-diagnosis of diseases. Given the developing nature of NMT tools, this can pose safety risks to multicultural communities with limited bilingual skills, low education, and low health literacy. Research is needed to scrutinise the reliability, credibility, usability of automatically translated patient health information.

**Objective:** We aimed to develop high-performing Bayesian machine learning classifiers to assist clinical professionals and healthcare workers in assessing the quality and usability of NMT on depressive disorders. The tool did not require any prior knowledge from frontline health and medical professionals of the target language used by patients.

**Methods:** We used Relevance Vector Machine (RVM) to increase generalisability and clinical interpretability of classifiers. It is a typical sparse Bayesian classifier less prone to overfitting with small training datasets. We optimised RVM by leveraging automatic recursive feature elimination and expert feature refinement from the perspective of health linguistics. We evaluated the diagnostic utility of the Bayesian classifier under different probability cut-offs in terms of sensitivity, specificity, positive and negative likelihood ratios against clinical thresholds for diagnostic tests. Finally, we illustrated interpretation of RVM tool in clinic using Bayes' nomogram.

**Results:** After automatic and expert-based feature optimisation, the best-performing RVM classifier (RVM_DUFS12) gained the highest AUC (0.8872) among 52 competing models with distinct optimised, normalised features sets. It also had statistically higher sensitivity and specificity compared to other models. We evaluated the diagnostic utility of the best-performing model using Bayes' nomogram: it had a positive likelihood ratio (LR+) of 4.62 (95% C.I.: 2.53, 8.43), and the associated posterior probability (odds) was 83% (5.0) (95% C.I.: 73%, 90%), meaning that approximately 10 in 12 English texts with positive test are likely to contain information that would cause clinically significant conceptual errors if translated by Google; it had a negative likelihood ratio (LR-) of 0.18 (95% C.I.: 0.10,0.35) and associated posterior probability (odds) was 16% (0.2) (95% C.I: 10%, 27%), meaning that about 10 in 12 English texts with negative test can be safely translated using Google.

## 1. Introduction

Neural machine translation technologies (NMT) are having increasing applications in diverse clinical settings in multicultural countries (1–4). They also facilitate the diagnosis of diseases for its convenience, wide availability, low-cost, and privacy of personal health information (5, 6). Research shows that despite the increased linguistic fluency and readability of NMT output in the target language of patients, accuracy, reliability, usability of automatically translated information remains a real challenge for the wide clinical uptake of this technology (7–10). However, there are increasing practical social needs for low-cost, versatile machine translation technologies such as Google Translate which can handle a very large number of well-studied or rare language pairs. In low-resource scenarios or public health emergencies, NMT applications are providing critical, timely language support to help reduce mortalities and the risks of transmission of highly infectious diseases when health professionals, frontline health workers engage with multicultural communities and populations, for whom adequately trained bilingual translators are not readily available.

Another important and increasingly common scenario of the public deployment of NMT tools is web-based self-diagnosis of diseases. For diverse reasons from personal preferences to concerns over individual privacy, translation technologies are providing more autonomy to multicultural users to make informed decisions about their health conditions, as well as enabling individual assessments of the need for more formal medical interventions or healthcare support. Many existing studies have explored, reported the use of NMT tools in facilitating the diagnosis of physical conditions and diseases such as at hospital emergency departments, whereas studies on the applicability of NMT technologies in the detection of mental health disorders are lacking (5, 9–11). Different from physical diseases, description of symptoms of mental disorders like anxiety, depression is more complex, subtle, ambiguous, subjective, susceptible to individual understanding, interpretation of the use of symptom related words, expressions. When translated to a distinct language, the meaning of symptoms of mental disorders in the source language can cause substantial confusion to users speaking a different target language or from a distinct cultural background.

## 2. Research Design

The purpose of our study was three-fold: first, we developed machine learning classifiers to support health professionals to detect, predict, and minimise the risks of using NMT tools in low-resource settings when engaging with non-English speaking vulnerable populations who might need urgent mental healthcare, interventions. After the development of the best-performing classifier, we provided detailed explanation of the application of the tool in the clinic to make probabilistic detection of English textual materials which may trigger clinically significant translation errors regarding the description of symptoms of depressive disorders if submitted to automatic translation tools. English source texts predicted by our Bayesian machine learning model as high-risk materials should be used with caution or be improved by health professionals with or without knowledge of the patients' language - a major advantage of our assessment tool. This relates to the second aim of our study.

A major strength of our Bayesian model was its interpretability, usability, adjustability in the context of multicultural health education and health information design. Machine learning algorithms are known for their complexity, difficulty to interpret. In dealing with classification problems, many popular machine learning models like random forest, support vector machine, neural networks, gradient boost tree can only provide hard, binary decisions. By contrast, Bayesian classifiers can capture the uncertainty in prediction, by providing the posterior probabilities of class membership, adjusting to varying prior probabilities and likelihood ratios (12, 13). In our study, combining automatic and expert-based feature optimisation techniques, we developed a sparse Bayesian machine classification model with a small number of features, mostly very easy-to-quantify linguistic features. This means that in health information review and revision, we can modify in a targeted manner a handful of linguistic features of the original English health materials, to significantly improve the suitability of English input materials for their automatic translation to patients' languages. This can be achieved through controlling, reducing the probabilities of predefined types of errors in automatic translation output—in our study, this refers to errors related to depression symptoms—under very low levels (with sensitivities above 85 or 95%). In Discussion, we elaborated on interpretation of our classifier using Bayes' nomogram (14–16) which provides posteriors, positive, negative likelihood ratios of the model prediction with different probability cut-offs.

Third, our assessment tool provides much-needed reassurance or research-based protection to online health information seekers who rely to varying extent on translated health materials. Having greater, safer access to quality translated English mental health materials can empower, support individuals from disadvantaged social backgrounds, for example, immigrants living in multicultural societies with limited English proficiency, low health literacy, lack of access to English medical and healthcare services. We trust that the safety function of high-performing machine learning classifiers to automatically detect, predict and label ‘risky' source texts does have the potential to be integrated into online NMT tools to help increase the public awareness of the pros and cons, that is, the convenience vs. the risk of online NMT tools in specialised medical and healthcare domains.

Last but not least, although we used the English and its matching back translation from Chinese pairs to develop classifiers, the methods we developed can be easily adapted for testing and validation with other language combinations. We used easy-to-quantify linguistic features of English materials and avoided the linguistic annotation of translated materials in the target languages, bearing in mind that for some minority languages, natural language processing techniques may not be available or reliable, which might limit the application of new tools as extensions or adaptations to our models in low-resource scenarios for minority communities and populations.

## 3. Materials

### 3.1. Data Collection and Screening Strategies

Table 1 lists the websites of major mental health organisations, federal or state agencies promoting mental health in major English-speaking countries: the UK, the U.S., Australia, Canada. We selected a large number (around 40) of websites of health authorities to collect varieties of patient-oriented, instead of professional level mental health resources in English, with a specific focus on the diagnosis and treatment of depressive disorders including clinical depression, melancholia, psychotic depression, antenatal/postnatal depression, and suicidal ideation prevalent among young people. Because of our strict selection criteria in terms of topical relevance and user-orientation (patient oriented instead of medical professionals), the final number of eligible full-length articles we collected after manual screening was 337, which equals to an average of 9 article per website.

**Table 1:** List of data collection websites.

**Websites URLs**			
1	https://americanmigrainefoundation.org	20	https://www.childline.org.uk
2	https://au.reachout.com	21	https://www.drugs.com
3	https://ccsmh.ca	22	https://www.healthline.com
4	https://familydoctor.org/condition/depression/	23	https://www.heretohelp.bc.ca
5	https://foundrybc.ca	24	https://www.independentage.org
6	https://headspace.org.au	25	https://www.mayoclinic.org
7	https://jack.org	26	https://www.menshealthforum.org.uk
8	https://kidshelpphone.ca	27	https://www.mentalhealth.org.uk
9	https://medlineplus.gov	28	https://www.mind.org.uk
10	https://mindyourmind.ca	29	https://www.msdmanuals.com
11	https://my.clevelandclinic.org	30	https://www.nami.org
12	https://patient.info	31	https://www.nimh.nih.gov
13	https://psychcentral.com	32	https://www.papyrus-uk.org
14	https://riseabove.org.uk	33	https://www.verywellmind.com
15	https://www.ageuk.org.uk	34	https://www.webmd.com
16	https://www.apa.org	35	https://www.womenshealth.gov
17	https://www.betterhealth.vic.gov.au	36	https://youngmenshealthsite.org
18	https://www.beyondblue.org.au	37	https://youngminds.org.uk
19	https://www.blackdoginstitute.org.au		

### 3.2. Labelling of Positive and Negative Cases

After collecting eligible full-length original English articles on depressive disorders, we submitted these articles for automatic translation to simplified Chinese using the Google Translate API. To assess the acceptability of the semantic distance between the original English texts and their automatically translated materials, we produced the backtranslations in English. We then conducted pairwise comparison of the original English and its matching backtranslation from Chinese with support of qualified bilingual researchers in our multi-institutional team. Based on the assessment of text pairs containing the source English text and its backtranslation, we labelled as positive cases original English mental health texts associated with backtranslations containing human verified errors of depression symptoms; we labelled as negative cases original English texts associated with backtranslations not containing clinically significant errors. In the evaluation process, human judgement on the acceptability of automatic translation errors was critical. To ensure labelling consistency, we assessed the inter-rater reliability between two trained researchers with professional English Chinese translation backgrounds, Cohen's kappa was generated using SPSS (v. 26): 0.864 (95% confidential interval: 0.834, 0.894). Discrepancies between two assessors was resolved by adjudication of a third trained researcher with advanced bilingual skills.

Table 2 offers illustrative examples of translation errors introduced by the NMT tool, and the level of severity of such errors which we considered not acceptable for clinical settings or online self-diagnosis of depressive disorders among online health information seekers. Using Google Translate, we found the word “depression” was consistently translated as “frustration” to Chinese, the word “guilt” was translated as “shame” in Hindi, and the word “judgemental” was translated as “critical” in Spanish. For users without bilingual skills like professional translators to evaluate the authenticity of automatic translation, or without health literacy like doctors, using NMT tools to assess or diagnose mental health conditions can be misleading, causing self-stigmatisation, discriminative attitudes or behaviours toward individuals exhibiting mild to severe mental disorder symptoms.

**Table 2:** Examples of diagnosis related errors in NMT outputs.

	**Original english material**	**Backtranslation from simplified chinese using GT**
1	A low mood that doesn't lift may be a sign of depression. However, depression isn't just about low mood. You may experience the following: Feeling bored or ***restless***.	Low mood that doesn't get better may be a sign of depression. However, depression is more than just a low mood. You may encounter the following situations: Feeling bored or ***upset***.
2	Harassment is behaviour which frightens you and causes you ***distress or alarm***.	Harassment is behaviour that scares you and causes you ***pain or panic***.
3	Depression can change how you feel physically, your thinking, your emotions, and your behaviours: ***Irritability and restlessness*** ***Aggressive***, abusive, or controlling behaviour.	Depression can change your body's feelings, ways of thinking, emotions, and behaviours: ***Anxious*** ***Offensive***, abusive, or controlling behaviour.
4	The ***Physical*** Effects of Depression: ***Psychomotor*** ***High blood pressure*** ***Medication side effects*** ***Coping***.	The ***Physiological*** Effects of Depression: ***Mental movement*** ***Hypertension*** ***Drug side effects*** ***Response***
5	Clinical depression is marked by a ***depressed mood***: Impaired concentration, indecisiveness Insomnia or ***hypersomnia*** (excessive sleeping) Restlessness or ***feeling slowed down*** Recurring thoughts of death or suicide	Clinical depression is characterised by ***low mood***: ***Inattention, indecision*** Insomnia or ***too much sleep*** (too much sleep) Fidgeting or ***feeling slow*** Repeated thoughts of death or suicide

## 4. Methods

### 4.1. Development of Machine Learning Classifiers

We divided the whole dataset (337) including 167 negative and 170 positive cases into 70% training and 30% testing datasets (as shown in Table 3), to develop machine learning classifiers to predict the probabilities of original English health texts which will contain clinically significant errors or not if the text were submitted to automatic translation. This new tool we developed aimed to offer a needed layer of protection to vulnerable online health information users more exposed to misdiagnosis of mental health conditions. The training dataset contained 118 negative and 117 positive cases used for developing machine learning classifiers. To reduce biases of classifiers, we deployed five-fold cross-validation. Specifically, we divided the entire training dataset into five folds of approximately equal sizes. We used four folds of data to develop classifiers and repeated the same procedure 5 times. We then calculated the mean area under the curve (AUC) of receiver operator characteristic (ROC) and its standard deviation on the training dataset. We tested the classifiers on the remaining 30% testing data to verify their performance.

**Table 3:** Data distribution of training and testing dataset.

**Datasets**	**Negative cases**	**Positive cases**
Training dataset	118	117
Testing dataset	49	53
Total	167	170

*Negative cases: original English texts not associated with back-translations containing clinically significant mistakes*.

*Positive cases: original English texts associated with back-translations containing clinically significant mistakes*.

### 4.2. Feature Annotation

In order to develop machine learning classifiers which are easy to use and interpret by the end users such as medical and health professionals, we chose to annotate the original English health texts with widely accessible, low-cost, or free English automatic annotation tools. Specifically, we selected three sets of English text annotation tools: dispersion rates based on lexical statistics from the British National Corpus (BNC); English semantic annotation system (USAS) developed by Lancaster University, UK; and automatic annotation of multiple English lexical, syntactic, structural features with Readability Studio (Oleander software).

#### 4.2.1. Lexical Dispersion Features (DiSp and DiWr) (20 in Total)

Lexical dispersion rate refers to the range of distribution of a word in spoken and written materials of the BNC corpus (17–19). Lexical dispersion rates are counted at 0.1 intervals such as DiSp1: 0–0.1, DiSp2: 0.1–0.2, DiWr1: 0–0.1, and DiWr2: 0.1–0.2. The higher the dispersion rate, the more widely distributed a word in spoken or written English. Dispersion rates thus serve as an indicator of the familiarity and general nature of lexis. It differs from frequency statistics, as words of high frequencies of occurrence may still have low dispersion rates, for example, domain-specific words used in certain professions, but still relatively unknown to the general public. We purposefully included English lexical dispersion rates as candidate features for developing machine learning classifiers, as we observed during the assessment of the acceptability of automatic translation options that common, familiar words seemed to cause automatic translation errors more often than specialised terms from the medical domain.

#### 4.2.2. English Semantic Features (USAS) (115 in Total)

The annotation system USAS provided an extensive coverage of the semantic classes of English words. The system was based on the Tom McArthur's Longman Lexicon of Contemporary English (20, 21). USAS has as many as 21 large classes and over a hundred sub-classes of semantic tags including general and abstract terms (A1-A15, 15 features); the body and the individual (B1-B5, 5 features); arts and crafts (C1); emotion (E1-E6, 6 features); food and farming (F1-F4, 4 features); government and public (G1-G3, 3 features); architecture, housing and the home (H1-H5, 5 features); money and commerce in industry (I1-I4, 4 features); entertainment, sports and games (K1-K6, 6 features); life and living things (L1-L3, 3 features); movement, location, travel and transport (M1-M8, 8 features); numbers and measurements (N1-N6, 6 features); substances, materials, objects and equipment (O1-O4, 4 features); education (P1), language and communication (Q1-Q4, 4 features); social actions, states and processes (S1-S9, 9 features); time (T1-T4, 4 features); world and environment (W1-W5, 5 features); psychological actions, states and processes (X1-X9, 9 features); science and technology (Y1-Y2, 2 features); grammar (Z0-Z9, Z99, 11 features) (22–24).

#### 4.2.3. Structural Features (STF) (24 in Total)

Readability Studio added 24 natural language features regarding morphological, lexical and syntactic complexity of the texts: average number of sentences per paragraph, number of difficult sentences (more than 22 words), average sentence length, number of interrogative sentences, number of exclamatory sentences, average number of characters, average number of syllables, number of numerals, number of proper nouns, number of monosyllabic words, number of unique monosyllabic words, number of complex (3+ syllable) words, number of unique 3+ syllable words, number of long (6+ characters) words, number of unique long words, misspellings, repeated words, wording errors, redundant phrases, overused words (x sentence), wordy items, Cliché, passive voice, sentences that begin with conjunctions (25–29).

### 4.3. Classifier Optimisation

**Figure 1:**
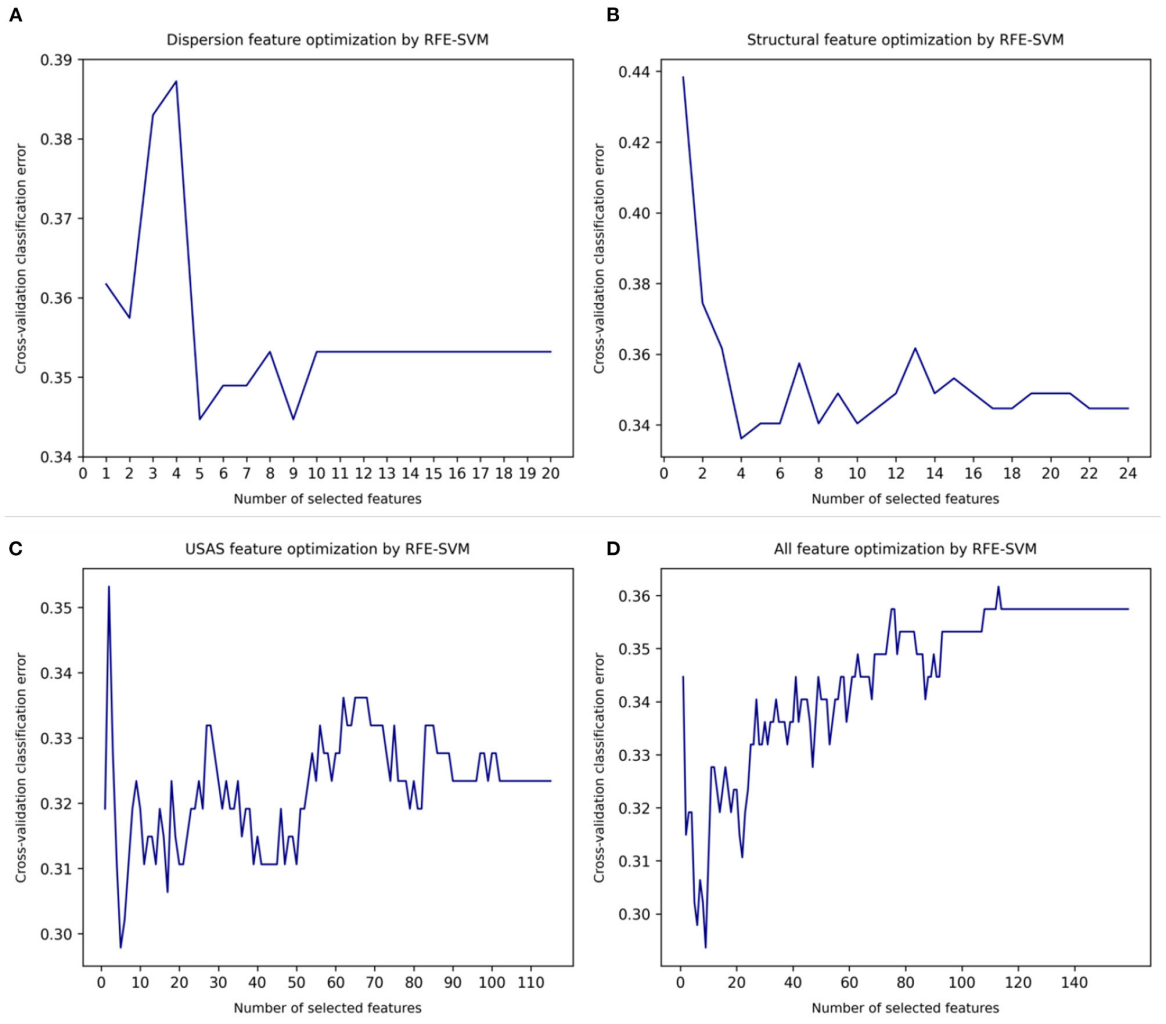
Automatic feature optimisation. **(A)** Lexical dispersion rate feature optimization. **(B)** Structural features optimization. **(C)** English semantic feature optimization. **(D)** Joint optimization of all features.

Figure 1 shows the automatic feature optimisation using recursive feature elimination with SVM as base estimator (RFE-SVM) (30, 31). Relevance vector machine (RVM) is a sparse classifier, and its performance improves significantly with less rather than more features to draw the surface between binary outcomes (12, 32, 33). We first applied separate optimisation on the 3 sets of features: lexical dispersion rate features (in total 20 features), structural features (in total 24 features), semantic features (in total 115 features). An optimised number of features was obtained when the minimal cross-validation classification error (CVCE) was reached. The optimised numbers of the 3 feature sets were: 5 for lexical dispersion rates (CVCE = 0.345) (Figure 1A); 4 for structural features (CVCE = 0.336) (Figure 1B), and 5 for English semantic features (CVCE = 0.298) (Figure 1C). Next, we applied a joint optimisation of all three features sets which reached an optimised feature set of 9 (CVCE = 0.294) (Figure 1D). Items in the optimised feature of lexical dispersion were DiSp7:0.6–0.7, DiSp8:0.7–0.8, DiSp9:0.8–0.9, DiWr9:0.8–0.9, and DiWr10:0.9–1.0. These confirmed our initial hypothesis that general, more familiar words were more likely to cause clinically significant errors in automatic translation output. Items in the optimised feature of structural features were number of complex (3+ syllable) words, number of unique, complex (3+ syllable) words, number of long (6+ characters) words, number of unique long words. Given that one English syllable contains at least 2 characters or letters, the first two and the second two features resembled each other.

In the following process, we fine-tuned this feature set by experimenting with alternative combinations of these 4 structural features. It shows that the most effective combination was number of unique 3+ syllable words and number of long (6+ characters) words (see Tables 4, 5). Lastly, items retained in the optimised semantic feature set were abstract terms denoting importance or significance and abstract terms denoting noticeability or markedness (A11); general or abstract terms denoting (propensity for) change, and general or abstract terms denoting causal relationship, or lack of it (A2); terms relating to the (state of the) physical condition (B2); terms depicting (level of) happiness and terms depicting (level of) contentment (E4); functional words such as prepositions, adverbs, conjunctions (Z5). It is useful to notice that the two sets of structural and semantic features were recurring neighbouring words of original, general English expressions which resulted in non-acceptable or clinically consequential automatic translations of depressive symptoms and conditions. These features were leveraged as important textual clues by machine learning classifiers to predict the likelihood of an English text of causing misunderstanding of depressive symptoms if the original content were translated with machine translation tools without a final quality check.

**Table 4:** The best-performing feature set DUSF12.

Best performing feature set DUSF12	Lexical dispersion rates	DiSp7:0.6-0.7, DiSp8:0.7-0.8, DiSp9:0.8-0.9, DiWr9:0.8-0.9, DiWr10:0.9-1.0
	Structural features	number of unique 3+ syllable words, number of long (6+ characters) words
	English semantic features	importance (A11),affect (A2),health and disease (B2),happy or sad (E4),grammatical bin (Z5)

**Table 5:** Data distribution of training and testing dataset.

**Feature set No**.	**RVM**	**Training data**		**Testing data**			
		**Mean AUC**	**STD**	**AUC**	**Accuracy**	**Sensitivity**	**Specificity**
FS1	**Full feature set (English lexical dispersion rates: Disp)**						
	Disp_20	0.693	0.059	0.769	0.696	0.623	0.776
	Disp_20 (Min-Max normalisation: MMN)	0.639	0.057	0.779	0.706	0.642	0.776
	Disp_20 (*L*_2_ normalisation: L2N)	0.659	0.08	0.738	0.686	0.717	0.653
	Disp_20 (Z-score normalisation: ZSN)	0.638	0.083	0.705	0.706	0.566	0.857
FS2	**Automatically optimised feature set (English lexical dispersion rates: Disp)**						
	Disp_5	0.679	0.06	0.767	0.667	0.566	0.776
	Disp_5 (MMN)	0.673	0.068	0.78	0.706	0.566	0.857
	Disp_5 (L2N)	0.652	0.062	0.715	0.686	0.736	0.633
	Disp_5 (ZSN)	0.679	0.068	0.787	0.735	0.642	0.837
FS3	**Full feature set (English structural feature: STF)**						
	STF_24	0.705	0.091	0.844	0.765	0.698	0.837
	STF_24 (MMN)	0.684	0.112	0.789	0.726	0.679	0.776
	STF_24 (L2N)	0.707	0.097	0.833	0.755	0.811	0.694
	STF_24 (ZSN)	0.702	0.126	0.798	0.745	0.717	0.776
FS4	**Automatically optimised feature set (English structural feature: STF)**						
	STF_4	0.699	0.07	0.852	0.745	0.623	0.878
	STF_4 (L2N)	0.699	0.071	0.813	0.745	0.604	0.898
	STF_4 (ZSN)	0.703	0.088	0.77	0.726	0.811	0.633
	STF_4 (ZSN)	0.706	0.067	0.811	0.745	0.604	0.898
FS5	**Full feature set (English semantic classes: USAS)**						
	USAS_115	0.718	0.05	0.849	0.765	0.774	0.755
	USAS_115 (MMN)	0.693	0.052	0.739	0.686	0.698	0.674
	USAS_115 (L2N)	0.677	0.073	0.755	0.647	0.604	0.694
	USAS_115 (ZSN)	0.69	0.057	0.788	0.706	0.66	0.755
FS6	**Automatically optimised feature set (English semantic classes: USAS)**						
	USAS_5	0.753	0.009	0.861	0.784	0.736	0.837
	USAS_5 (MMN)	0.727	0.042	0.841	0.775	0.717	0.837
	USAS_5 (L2N)	0.713	0.044	0.711	0.706	0.679	0.735
	USAS_5 (ZSN)	0.738	0.035	0.832	0.784	0.736	0.837
FS7	**Full feature set (Including disp, STF, and USAS)**						
	Disp_20 + STF _24 + USAS_115 = F159	0.69	0.08	0.845	0.804	0.793	0.816
	F159 (MMN)	0.668	0.065	0.714	0.657	0.642	0.674
	F159 (L2N)	0.74	0.06	0.841	0.765	0.774	0.755
	F159 (ZSN)	0.707	0.028	0.778	0.716	0.66	0.776
FS8	**Automatically optimised full feature set (Including disp, STF and USAS)**						
	DSUF9	0.693	0.05	0.872	0.824	0.774	0.878
	DSUF9 (MMN)	0.727	0.082	0.838	0.794	0.774	0.816
	DSUF9 (L2N)	0.747	0.024	0.798	0.726	0.736	0.714
	DSUF9 (ZSN)	0.733	0.052	0.838	0.765	0.642	0.898
FS9	**Combinations of separately optimised feature Sets**						
	Disp_5 + USAS_5 = DUF10	0.742	0.016	0.869	0.804	0.774	0.837
	DUF10 (MMN)	0.739	0.06	0.799	0.706	0.66	0.755
	DUF10 (L2N)	0.777	0.02	0.862	0.775	0.793	0.755
	DUF10 (ZSN)	0.725	0.065	0.795	0.677	0.642	0.714
FS10	Disp_5 + STF_4 = DSF9	0.698	0.075	0.845	0.735	0.585	0.898
	DSF9 (MMN)	0.684	0.085	0.808	0.716	0.547	0.898
	DSF9 (L2N)	0.726	0.073	0.842	0.745	0.793	0.694
	DSF9 (ZSN)	0.697	0.076	0.782	0.726	0.566	0.898
FS11	USAS_ 5 + STF_4 = USF9	0.722	0.062	0.882	0.843	0.793	0.898
	USF9 (MMN)	0.741	0.06	0.841	0.745	0.736	0.755
	USF9 (L2N)	0.75	0.033	0.806	0.716	0.736	0.694
	USF9 (ZSN)	0.736	0.066	0.851	0.775	0.811	0.735
FS12	Disp_5 + USAS_5 + STF_4 = DUSF14	0.709	0.057	0.882	0.833	0.811	0.857
	DUSF14 (MMN)	0.742	0.041	0.763	0.677	0.623	0.735
	DUSF14 (L2N)	0.747	0.042	0.877	0.814	0.868	0.755
	DUSF14 (ZSN)	0.741	0.042	0.801	0.677	0.642	0.714
FS13	Disp_5 + USAS_5 + STF_2 = DUSF12	0.709	0.038	0.887	0.804	0.717	0.898
	DUSF12 (MMN)	0.745	0.076	0.79	0.667	0.604	0.735
	DUSF12 (L2N)	0.773	0.017	0.887	0.833	0.849	0.816
	DUSF12 (ZSN)	0.734	0.072	0.809	0.696	0.66	0.735

## 5. Results

Table 5 shows the result of the performance of relevance vector machine (RVM) on the training and the testing datasets. To identify the best performing classifier, we first compared 4 pairs of original feature sets and their optimised feature sets: lexical dispersion rates (FS1 and FS2), structural features (FS3 and FS4), semantic features (FS5 and FS6), as well as the full feature set of the three and its optimised version (FS7 and FS8). Within each feature set, we also tested the impact of different feature normalisation techniques on the model performance. We used three common normalisation methods which were max-min normalisation (MMN), *L*_2_ normalisation (L2N) and Z-score based normalisation (ZSN). The results show that overall, feature optimisation improved the classifier performance on the testing data, while reducing the total number of features significantly, helping to develop more parsimonious, effective, and interpretable models. For example, the optimised feature set of lexical dispersion rates (5 features) achieved a similar area under the curve of receiver operator characteristic (AUC of ROC) 0.767 when compared to that of the original feature set of lexical dispersion rates (20 features) (0.769). The optimised feature set of structural features (4 features) achieved a higher AUC (0.852) when compared to that of the original feature set of structural features (24 features) (0.844). The optimised feature set of semantic features (5 features) also reached a higher AUC (0.861) when compared to the AUC of the original semantic feature set (115 features in total) (0.849). To reinforce this pattern, the AUC of the optimised full feature set (9 features) (0.872) was improved over that of the original, non-optimised full feature set (containing 159 features) (0.845). The impact of different normalisation techniques on the optimised feature sets was mixed.

**Figure 2:**
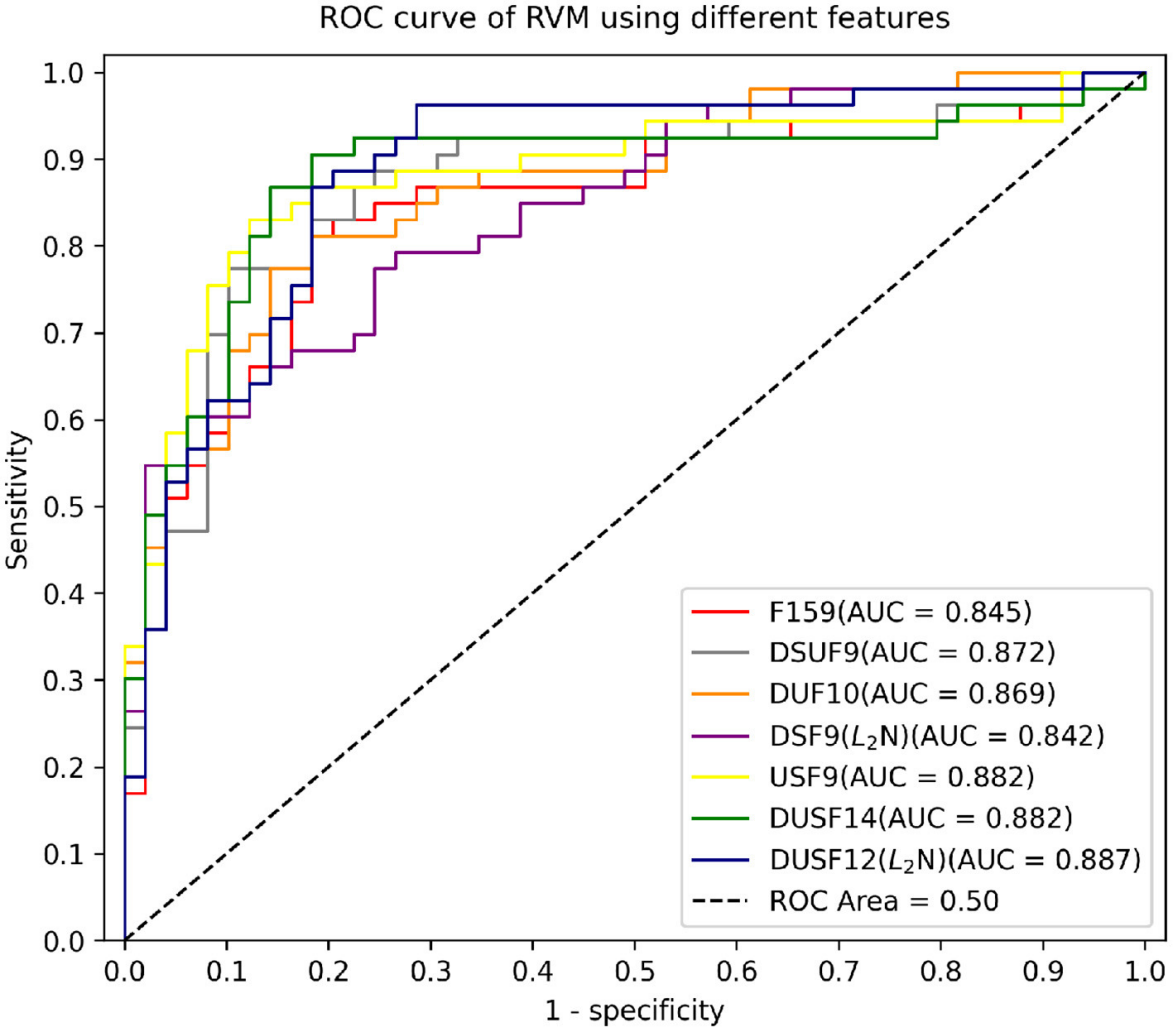
AUC of RVMs on testing data using different feature sets.

An important limitation of these separately and jointly optimised feature sets was their lack of balanced performance in terms of model sensitivity and specificity - some classifiers had higher sensitivity but lower specificity (for example, Disp_5 (L2N), STF_4 (ZSN), and USAS_5) and some had higher specificity but lower sensitivity [for example, Disp_5 (MMN), Disp_5 (ZSN), STF_4 (L2N), STF_4 (ZSN), USAS_5 (ZSN), USAS_5 (MMN), and DSUF9 (ZSN)]. To identify machine classifiers with both high sensitivity, specificity, especially higher sensitivity, we compared the performance of RVMs with different combinations of optimised feature sets as shown in rows of FS9-FS13. We experimented with five combinations of optimised feature sets: FS9 was the combination of optimised lexical dispersion and optimised semantic feature sets (DUF10). FS10 was the combination of the optimised lexical dispersion and the optimised structural features (DSF9). FS11 was the combination of the optimised semantic and the optimised structural features (USF9). FS12 was the combination of the three optimised feature sets (DUSF14), and FS13 was a simplified version of DUSF14 by leaving out two structural features that were very similar to those remained the model. Normalisation boosted the performance of classifiers on some occasions. Figure 2 shows the top 7 classifiers with the highest AUC among the 52 competing models. These were (in order of increasing AUC) DSF9 (L2N) (AUC = 0.842), F159 (AUC = 0.845), DUF10 (AUC = 0.869), DSUF9 (AUC = 0.872), USF9 (AUC = 0.882), DUSF14 (AUC = 0.882) and finally, the best-performing classifier DUSF12 (AUC = 0.887).

Tables 6, 7 show the paired sample *t*-tests of the sensitivity and specificity between the best-performing classifier DUSF12 with *L*_2_ features normalisation with the other 6 top classifiers. To reduce the rates of false discovery and false omission in multiple comparison, we adjusted the two-sided significance level (*p* = 0.05) using Benjamini-Hochberg correction procedure. In Tables 6, 7, pairs of classifiers were ranked in order of the increasing *p*-values: pairs with small *p*-values were ranked lower and assigned lower Benjamini-Hochberg critical values, using the formula (i/m)Q, whereas i was the individual *p*-value's rank, m was the total number of tests, Q was the false discovery rate (one minus specificity) set at 0.05. The results show that the best-performing DUSF12 had not only the highest AUC on the testing dataset, but also statistically higher sensitivity than the other six classifiers. In terms of specificity, DUSF12 had statistically similar specificity to that of the un-optimised, full feature set (F159), despite that it only had 12 features (shown in Table 4). The specificity of DUSF12 however was statistically lower than that of other classifiers. Considering the importance of model sensitivity to assess the suitability or the lack of it of classifiers for automatic translations, we retained the DUSF12 model as our best-performing classifier.

**Table 6:** Paired sample *t*-test of the difference in sensitivity between the best model with other models.

**No**.	**Pairs of RVMs**	**Mean difference**	**S.D**.	**95% Confidence interval of Difference**		**P-value**	**Rank**	**(i/m)Q**	**Sig**.
				**Lower**	**Upper**				
1	DUSF12 (L2N) vs. DSUF9	0.0755	0.0163	0.0435	0.1074	0.0152	1	0.0083	**
2	DUSF12 (L2N) vs. DUF10	0.0755	0.0163	0.0435	0.1074	0.0152	2	0.0167	**
3	DUSF12 (L2N) vs. F159	0.0566	0.0128	0.0315	0.0817	0.0166	3	0.025	**
4	DUSF12 (L2N) vs. DSF9(L2N)	0.0566	0.0128	0.0315	0.0817	0.0166	4	0.0333	**
5	DUSF12 (L2N) vs. USF9	0.0566	0.0128	0.0315	0.0817	0.0166	5	0.0417	**
6	DUSF12 (L2N) vs. DUSF14	0.0377	0.009	0.0202	0.0553	0.0183	6	0.05	**

**Table 7:** Paired sample t test of the difference in specificity between the best model with other models.

**No**.	**Pairs of RVMs**	**Mean difference**	**S.D**.	**95% Confidence interval of Difference**		**P-value**	**Rank**	**(i/m)Q**	**Sig**.
				**Lower**	**Upper**				
1	DUSF12 (L2N) vs. DSF9(L2N)	0.1225	0.0206	0.082	0.1629	0.0093	1	0.0083	^**^
2	DUSF12 (L2N) vs. DUF10	–0.0204	0.0049	–0.0301	–0.0107	0.0189	2	0.0167	^**^
3	DUSF12 (L2N) vs. DUSF14	–0.0408	0.0104	–0.0613	–0.0203	0.0211	3	0.025	^**^
4	DUSF12 (L2N) vs. DSUF9	–0.0612	0.0166	–0.0938	–0.0286	0.0238	4	0.0333	^**^
5	DUSF12 (L2N) vs. USF9	–0.0816	0.0237	–0.128	–0.0352	0.0269	5	0.0417	^**^
6	DUSF12 (L2N) vs. F159	0	0	0	0	1	6	0.05	

** *Statistical significance at 0.05 level using Benjamini-Hochberg correction procedure*.

## 6. Discussions

**Figure 3:**
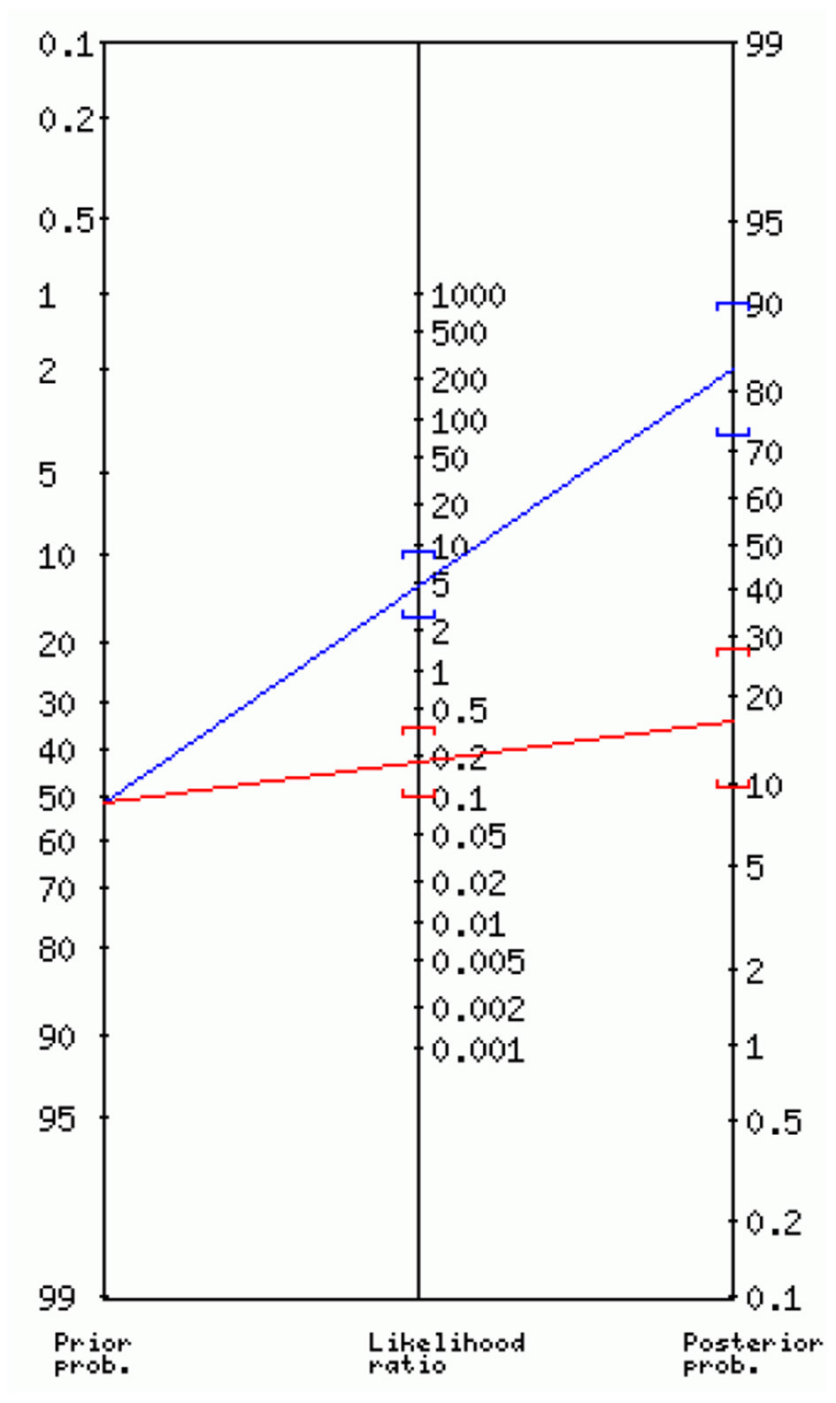
Interpreting the diagnostic utility of the best-performing classifier using Bayes' nomogram.

To illustrate the practical use of the classifier, Bayes' nomograph was used to demonstrate diagnostic classifier characteristics including sensitivity, specificity, likelihood ratios, and the post-test probability of positive cases given the pre-test probability and classifier characteristics (14–16, 34–37). In Figure 3, the axis on the left shows the prior or the baseline probability of the event of interest, which in our study was the prevalence of mistranslations introduced by neural machine translation tools of the original English patient health materials on depressive disorders including melancholia, psychotic, antenatal, postnatal depression, and suicidal ideation. It was currently at 52%. The middle axis represents likelihood or odds ratio which provide the estimate between rate ratios, which are affected by priors. Likelihood ratio can be positive or negative. A positive likelihood ratio (LR+) is the ratio between sensitivity (proportion of true positive cases) and false positivity (proportion of false positive cases, or one minus specificity). In our study, the best-performing classifier (RVM_DUSF12 with *L*_2_ normalisation) had a LR+ 4.62 (95% C.I.: 2.53, 8.43). If we use a straight edge on the nomogram, line up the prior (0.52) on the left axis, with the LR+ (4.62) on the middle axis, we can find the posterior probability on the right axis which was 83% (95% C.I.: 73%, 90%). The odds of the posterior probability of positive cases was 5.0, which means that around 10 in every 12 English health texts with a positive test result after screened by our classifier (over the 0.5 probability cut-off) will contain clinically significant errors if translated automatically. The middle axis can also be negative odds ratio which is the ratio between false negative cases (one minus sensitivity) and true negative cases. In our study, the negative likelihood ratio was 0.18 (95% C.I.: 0.10, 0.35). If repeating the same procedure of reading the Bayes' nomograph, we can find the posterior probability on the right axis which was 16% (95% C.I.: 10%, 27%). The odds of the posterior probability of true negative cases was 0.2, meaning that around 10 in every 12 English health texts with a negative test result after screened by our classifier (below the 0.5 probability cut-off) will not contain any clinically significant errors if translated automatically, and thus be translated safely using automatically tools.

High quality, accurate automatic health translation can facilitate the communication, diagnosis, detection, and early treatment of depressive disorders among vulnerable populations at risk. These include communities and populations with limited English proficiency, low education, and low health literacy levels. Automatic translations containing clinically significant errors can cause delays or mistreatment of mental disorders such as the variety of depressive disorders we studied. With the increasing quality, versatility, cost-effectiveness, and online accessibility of neural machine translation technologies, they have important potential for use in health and medical settings, especially in low-resource scenarios or health emergencies. However, at the current stage, our study of quantitative NMT output shows that the prevalence of clinically significant errors was still relatively high (about half of Chinese translations of English materials by Google Translate). Considering that the English and Chinese language pair is a well-studied translation combination, the rates of mistakes in other less-studied language pairs could be much higher, such as ethnic minority languages or aboriginal languages. There is a real need for developing research instruments and mechanisms to account for, detect and reduce the likelihood of clinically significant errors introduced in the automatic translation process. The primary function of the best-performing classifier (RVM_DUSF12 with *L*_2_ normalisation) we developed was to assist and support medical, health professionals working in low-resource scenarios, that is, without the support of trained bilingual health translators nor having any knowledge of the languages used by patients. The classifier we developed can support health professionals to optimise the complex, labour-intensive and highly skilled process of selecting, evaluating original English materials which can be effectively and safely translated with latest NMT tools to ensure the automatically translated materials are accurate, informative for the intended users. This can help reduce, or compensate for the costs of training, hiring bilingual health translators when the needs for such skilled workers are high, or very hard to source. Integrating high-precision, automatic evaluation tools such as the classifiers we developed can help design and deliver more sustainable, cost-effective mental healthcare services.

Our research shows that despite the existing issues of online NMT tools, their reliability, usability can be effectively predicted and mitigated by machine learning classifiers to reduce the risks of web-based diagnosis, treatment of severe mental disorders such as depression. The Bayesian machine learning classifiers we developed can support frontline health workers and professionals to make informed decision on the use of automatic translation tools to communicate with their patients and caregivers, which can be of important practical use in low-resource scenarios of depression diagnosis and treatment planning. The classifier we developed can be effectively adapted for other languages using available natural language systems.

## Data Availability Statement

The raw data supporting the conclusions of this article will be made available by the authors, without undue reservation.

## Author Contributions

MJ responsible for project administration. MJ and WX were responsible for the study concept and design. MJ drafted the manuscript. WX was responsible for the machine learning model development and data analysis. XQ, MZ, FY, and TZ were responsible for the data acquisition and annotation. C-YC, K-YL, and TH supervised the machine learning model development. All authors provided critical feedback and helped shape the analysis, research, and full manuscript. All authors contributed to the article and approved the submitted version.

## Funding

The work is supported by grants from the National Natural Science Foundation of China (No. 61772146) and Natural Science Foundation of Guangdong Province (No. 2021A1515011339).

## Conflict of Interest

The authors declare that the research was conducted in the absence of any commercial or financial relationships that could be construed as a potential conflict of interest.

## Publisher's Note

All claims expressed in this article are solely those of the authors and do not necessarily represent those of their affiliated organizations, or those of the publisher, the editors and the reviewers. Any product that may be evaluated in this article, or claim that may be made by its manufacturer, is not guaranteed or endorsed by the publisher.
